# Seasonality and Co‐Detection of Respiratory Viral Infections Among Hospitalised Patients Admitted With Acute Respiratory Illness—Valencia Region, Spain, 2010–2021

**DOI:** 10.1111/irv.70017

**Published:** 2024-10-22

**Authors:** George Shirreff, Sandra S. Chaves, Laurent Coudeville, Beatriz Mengual‐Chuliá, Ainara Mira‐Iglesias, Joan Puig‐Barberà, Alejandro Orrico‐Sanchez, Javier Díez‐Domingo, Valencia Hospital Surveillance Network for the Study of Influenza and Other Respiratory Viruses (VAHNSI), Lulla Opatowski, F. Xavier Lopez‐Labrador

**Affiliations:** ^1^ Epidemiology and Modelling of Antibiotic Evasion (EMAE), Institut Pasteur Université Paris Cité Paris France; ^2^ Anti‐Infective Evasion and Pharmacoepidemiology Team Université Paris‐Saclay, UVSQ, Inserm, CESP Montigny‐Le‐Bretonneux France; ^3^ New Products & Innovation Sanofi Vaccines Lyon France; ^4^ Virology Laboratory, Genomics and Health Area Fundación para el Fomento de la Investigación Sanitaria y Biomédica de la Comunitat Valenciana (FISABIO‐Public Health) Valencia Spain; ^5^ CIBER‐ESP Instituto de Salud Carlos III Madrid Spain; ^6^ Vaccine Research Area Fundación para el Fomento de la Investigación Sanitaria y Biomédica de la Comunitat Valenciana (FISABIO‐Public Health) Valencia Spain; ^7^ Department of Microbiology & Ecology, Medical School University of Valencia Valencia Spain

**Keywords:** co‐detection, co‐infection, influenza, respiratory virus, rhinovirus, RSV respiratory syncytial virus, seasonality, severe acute respiratory syndrome coronavirus 2

## Abstract

**Background:**

Respiratory viruses are known to represent a high burden in winter, yet the seasonality of many viruses remains poorly understood. Better knowledge of co‐circulation and interaction between viruses is critical to prevention and management. We use > 10‐year active surveillance in the Valencia Region to assess seasonality and co‐circulation.

**Methods:**

Over 2010–2021, samples from patients hospitalised for acute respiratory illness were analysed using multiplex real‐time PCR to test for 9 viruses: influenza, respiratory syncytial virus (RSV), parainfluenza virus (PIV), rhino/enteroviruses (HRV/ENV), metapneumovirus (MPV), bocavirus, adenovirus, SARS‐CoV‐2 and non‐SARS coronaviruses (HCoV). Winter seasonal patterns of incidence were examined. Instances of co‐detection of multiple viruses in a sample were analysed and compared with expected values under a crude model of independent circulation.

**Results:**

Most viruses exhibited consistent patterns between years. Specifically, RSV and influenza seasons were clearly defined, peaking in December–February, as did HCoV and SARS‐CoV‐2. MPV, PIV and HRV/ENV showed less clear seasonality, with circulation outside the observed period. All viruses circulated in January, suggesting any pair had opportunity for co‐infection. Multiple viruses were found in 4% of patients, with more common co‐detection in children under 5 (9%) than older ages. Influenza co‐detection was generally observed infrequently relative to expectation, while RSV co‐detections were more common, particularly among young children.

**Conclusions:**

We identify characteristic patterns of viruses associated with acute respiratory hospitalisation during winter. Simultaneous circulation permits extensive co‐detection of viruses, particularly in young children. However, virus combinations appear to differ in their rates of co‐detection, meriting further study.

## 1 Introduction

It is widely recognised that in the temperate northern hemisphere, viral respiratory infections cause upsurges during winter. However the magnitude of this seasonality (amplitude, peak and duration) differs substantially between viruses [1]. Some viruses peak early in the winter season and some late, while others can have more than one distinct annual peak caused by different subtypes [2] or have larger peaks every other year [3]. Seasonality can be geographically specific, with lower latitudes associated with earlier peaks in influenza in the northern hemisphere [4]. These patterns have direct implications for burden of single infections, but interactions between viruses in co‐infection (simultaneous active infection of an individual with two viral species) may exacerbate or mitigate the risks associated with co‐circulation (simultaneous circulation of two viral species within the same population) of viruses.

Several mechanisms have been suggested for viral interactions, including inhibition of host immunity or cell fusion resulting in synergistic interaction and interference through host resource competition or stimulation of innate immunity through interferon resulting in competitive interaction [5]. Both synergistic and competitive interactions may modify either susceptibility to co‐infection, or its pathogenicity [6], which may lead to poorer clinical outcomes in patients [7, 8, 9, 10, 11]. All else being equal, synergistic interactions between a pair of viruses would increase the chance of co‐detection, while competitive interactions would decrease it.

While national level surveillance exists for Influenza and more recently RSV in many countries, data are generally not linked to clinical outcomes, and usually no surveillance data exist for other respiratory viruses for which seasonal patterns and epidemiological burden are less well described. Furthermore, only a few multi‐year studies have been able to address questions around interaction and co‐infection by testing for multiple respiratory viruses within the same sample [12, 13, 14, 15]. Designs of these studies included passive enrolment in general practice [12, 13] and passive enrolment across primary, secondary and tertiary healthcare [14, 15], with only two studies sampling over more than 2 years [14, 15]. A systematic active surveillance protocol to characterise all viruses in respiratory illness requiring hospitalisation is lacking: While some studies have conducted multiplex viral screening of samples from acute respiratory hospitalisation, these have been done only in children and not over multi‐year timescales [16, 17, 18, 19, 20, 21].

In this work, we analysed data collected through systematic winter surveillance in patients of all ages hospitalised in tertiary hospitals between 2010 and 2021 in the Valencia Region, Spain, to examine seasonality of individual respiratory viruses, and to explore the extent of single infections and co‐infections between pairs of viruses by examining numbers of observed co‐detections.

## 2 Methods

### 2.1 Study Population

The Valencia Hospital Surveillance Network for the Study of Influenza and Other Respiratory Viruses (VAHNSI) is an active surveillance network prospectively analysing respiratory hospitalisations in tertiary‐care public hospitals in the Valencia Region of Spain [22]. The study was conducted over 2010–2021 across 11 participating hospitals, of which an average of 5 hospitals participated each year.

**FIGURE 1:**
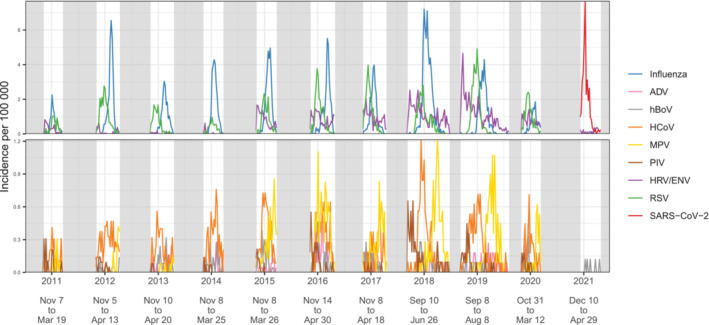
Observed seasonal curves for each virus in each season, per capita in the total catchment population. Each virus is shown in a different colour. The top panel shows the viruses with maximum incidence greater than 2 per 100,000 per week and the bottom those with lower maximum incidence. The greyed out areas are those in which data were not collected, and the text underneath the graph shows the dates of each data collection season. Each virus curve is displayed separately on a separate panel in Figure S1.

The sampling was generally conducted during the putative Influenza seasons (November to March inclusive) between mid‐2010 and mid‐2021, with the exceptions of 2017/18 and 2018/19 when the sampling season was deliberately extended for the purposes of the study (September to June in 2017/18 and September to August in 2018/19), and in 2020 when sampling was disrupted by the COVID‐19 pandemic (September to early March in 2019/20 and December to May in 2020/21). Specific dates of study for each season are shown at the bottom of Figure 1. Children under 18 were not included in 2010/11 but were included thereafter. Potential participants were those admitted to participating hospitals with acute respiratory illness. Dedicated nurses screened all hospitalised patients discharged from the Emergency Department if the referral was possibly related to a respiratory infection. To qualify for enrolment in the study, patients needed to be resident in the catchment area of one of the participating hospitals, non‐institutionalised, to have been admitted < 48 h before enrolment and not have been previously discharged from a hospital within 30 days prior to the current admission. Only patients with acute respiratory illness, having symptom onset < 7 days before hospital admission date, were considered for enrolment. The symptoms required for enrolment were age specific. Patients ≥ 5 years of age had to fit the European Centre for Disease Prevention and Control clinical case definition of influenza‐like illness, meaning they had at least one systemic symptom (fever or feverishness, headache, myalgia or malaise) and one respiratory symptom (cough, sore throat or shortness of breath). For children < 5 years, inclusion criteria was broad, including any clinical conditions potentially associated with a presentation of acute respiratory illness (list of conditions reported in Table A1). Clinical and demographic characteristics from patients were obtained by a face‐to‐face interview or by consulting medical records. Informed consent was taken before enrolment from patients or their legal guardians where appropriate [22].

Enrolled patients had oropharyngeal and nasopharyngeal swabs collected if aged ≥ 14 years or nasal and nasopharyngeal swabs if < 14 years. Each patient was sampled at a single time point. Both swabs were combined in one tube of viral transport media (Copan, Italy) and frozen at or below −20°C at the study site until shipped refrigerated to the coordinating site's centralised virology laboratory [22]. The dataset we worked with was aggregated by week and by age group. The size of the denominator population was the total estimated catchment population for each age group across all hospitals enrolled within each season.

### 2.2 Laboratory Analysis

One third of the viral transport medium volume (1 mL) was used for total nucleic acids extraction using an automated silica‐based method (Nuclisens Easy‐Mag, BioMérieux, Lyon, France). Subsequently, extracted nucleic acids were analysed using a real time multiplex reverse transcription polymerase chain reaction (RT‐PCR) panel, testing for the presence of adenovirus (ADV), human bocavirus (hBoV), human non‐SARS coronaviruses (229E, HKU1, NL63 and OC43, referred to as HCoV), human metapneumovirus (MPV), human parainfluenza viruses (1–4, referred to as PIV), respiratory syncytial virus (A/B, RSV), rhino/enteroviruses (HRV/ENV) and influenza viruses (A/B) [23, 24, 25, 26, 27, 28, 29]. Influenza viruses were further subtyped by real‐time RT‐PCR as A(H1N1pdm09) or A(H3N2) and by B lineage (Victoria or Yamagata) [24, 30]. From the start of the study to the 2013/14 season, the master‐mix AgPath‐ID One‐Step RT‐PCR Kit (Ambion, USA) was used, while from 2014/15 onwards, the master‐mix QScript XLT One‐Step RT‐qPCR ToughMix (Quantabio, MA, USA) was used. From 2014/15 onwards, the primers and probes were also updated for RSV (to include new RSV‐B circulating clades) [31] and for HRV/ENV (to increase sensitivity). Samples from September 2018 onwards were retrospectively tested for SARS‐CoV‐2 [32] and prospectively from December 2020. An external laboratory was used to conduct the analyses in the 2010/11 and 2011/12 seasons. A co‐detection sample is defined as one that is positive for more than one viral species.

### 2.3 Statistical Analysis

Week numbers were defined according to the International Organization for Standardization (ISO) 8601 format meaning that they begin on a Monday, and the week containing 1 January is week 1 if it has at least 4 days in the new year and otherwise forms the final week of the previous year. To allow comparison between seasons, we defined the final week of the year as 0 and weeks prior to this as negative numbers [33].

Confidence intervals for the proportion of co‐detections were represented by the Jeffreys interval, wherein a beta distribution with parameters *x + 0.5* and *n* − *x + 0.5*, where *n* is the number of patients testing positive for a given virus and *x* is the number of patients with co‐detection including that virus, is evaluated at probability 2.5% and 97.5%.

### 2.4 Expected Co‐Detection Incidence

For a given pair of viruses (*x* and *y*), the number of observed co‐detections wherein the patient's combined sample was positive for both viruses (${\mathrm{Inc}}_{x+y}$), was counted across all age groups *a* and weeks *t*.

$${\mathrm{Inc}}_{x+y}=\sum_{a}\sum_{t}{\mathrm{Inc}}_{x+y,a,t}$$

The number of incident cases with virus *x* (including co‐detections with *y*) in age group *a* and week *t* is given by ${\mathrm{Inc}}_{x,a,t}$, while the total number of samples in that age group *a* and week *t* is given by $T_{a,t}$. We defined the expected number of co‐detections for this pair for each age group *a* and week *t* (${\text{EInc}}_{x+y,a,t}$) based on a crude model of independent circulation as the product of the proportion of samples testing positive for each virus individually. This enabled us to account for the extent of co‐circulation within the same age group for each pair of viruses.

$$\;E{\mathrm{Inc}}_{x+y,a,t}=\frac{{\mathrm{Inc}}_{x,a,t}{\mathrm{Inc}}_{y,a,t}}{T_{a,t}}$$

Then the total expected number of co‐detections was summed across all age groups and weeks:

$${\text{EInc}}_{x+y}=\sum_{a}\sum_{t}{\text{EInc}}_{x+y,a,t}$$

The ratio of the total observed over total expected number of co‐detections was calculated for each pair of viruses.

$$\text{Observed}-\text{Expected Ratio}={\mathrm{Inc}}_{x+y}/{\text{EInc}}_{x+y}$$

Credibility intervals for the observed‐expected ratios were derived by bootstrapping analysis, in which the rows of raw data representing ${\mathrm{Inc}}_{x+y,a,t}$ and ${\text{EInc}}_{x+y,a,t}$ for each virus pair were resampled with replacement 1000 times, and the ratio recalculated. This was also repeated on a dataset from which the seasons before 2014/15 were excluded.

All analyses were conducted using *R* 4.2.0 [34], with plots created using *ggplot2*.

### 2.5 Ethics

The Ethics Research Committee of the Dirección General de Salud Pública‐Centro Superior de Investigación en Salud Pública (DGSP‐CSISP) approved the original protocol of the study, described in [22], and our analysis was based on this data in aggregate.

## 3 Results

Between 3 and 10 hospitals participated during each season, with an average of around 500 patients successfully enrolled in each participating hospital per season. A total of 62,265 patients across all seasons were approached for screening, of whom 59,237 (95%) consented to participate. Of these, 28,865 patients fulfilling the criteria for acute respiratory illness were sampled and successfully analysed, of whom 18,295 (63%) were negative for all viruses tested, 10,115 (35%) had a single virus detected and 455 (2%) had more than one virus detected (439 with two viruses, 16 with three viruses). An additional 120 enrolled patients with undetermined laboratory results were excluded from analysis. A single patient sample was lacking age data and was excluded from age‐specific analysis.

### 3.1 Seasonality of Viruses

The incidence curves for each virus over the studied period are shown overlaid in Figure 1, and on separate panels in Figure S1, revealing seasonal patterns characterising each pathogen. Recorded peaks were of consistent size for most viruses, although for MPV, PIV, HRV/ENV and RSV, the incidence was generally higher in the latter seasons of the study. Although incident rates differed substantially between age groups, there were no visual differences by age in the shapes of seasonal epidemics (Figure S2). Influenza virus appeared to generate a high peak every year, although when disaggregated over subtypes (Figure S3), Influenza B did not occur every year, and among Influenza A, often one of either A(H1N1pdm09) or A(H3N2) subtypes was dominant, with the exception of 2017/18 and 2018/19 seasons when both subtypes were detected at similar magnitude.

**FIGURE 2:**
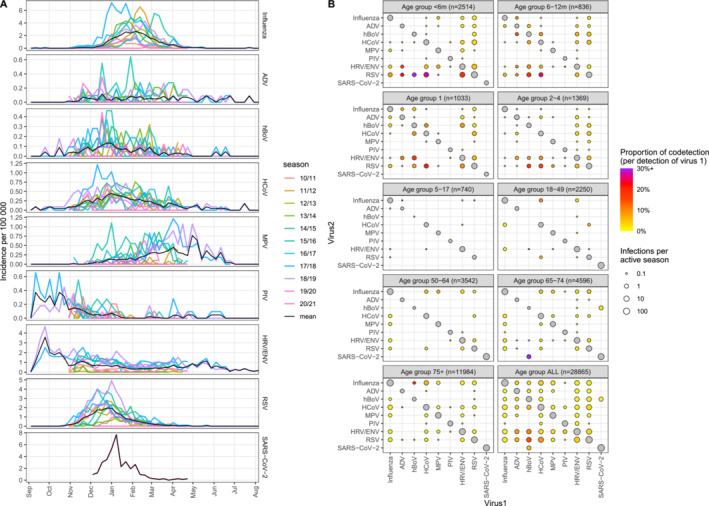
(A) Seasonality trends for each virus, with seasons overlaid. Each row is a different virus, with colours representing seasons, and the black line showing the weekly mean incidence per virus. (B) The number of mono‐detections and co‐detections by age group and all ages combined. Each panel represents an age category, with the panel label indicating both the age and number of patients with valid tests. Size of points indicates average number of detections per active season (SARS‐CoV‐2 was considered active for one season, all other viruses for 10 seasons in ages < 5 and 11 seasons otherwise) on a log scale. The diagonal represents the number of mono‐detections, while the off‐diagonal represent co‐detections, with colour representing the proportion of detections of Virus1 that are co‐detections (capped at 30%). The matrix is symmetrical for point size but not colour.

Seasonal trends were explored by overlaying the data from different years (Figure 2A). Influenza viruses peaked across January to March, but the exact timing of the peak was variable across seasons. RSV had a more consistent pattern peaking in December. Based only on a single season of data (2020/21), SARS‐CoV‐2 displayed a pronounced peak around January, while all other pathogens experienced very low incidence. MPV, PIV and HRV/ENV all appeared to exhibit high periods of transmission outside the studied follow up period. Based on the seasons where the study was extended (2017/18 and 2018/19), MPV showed high transmission late in the season (May–June), and PIV and HRV/ENV showed high transmission early in the season (September–October). We quantified general trends in the timing, amplitude and duration of seasonal outbreaks using seasonal waveform modelling [33] (Figure S4). Estimates of the timing of the peak were consistent for Influenza, RSV, and to some extent HCoV, but poor and characterised by high variability and large credibility intervals for other viruses. Crucially, all assayed viruses displayed substantial circulation in January, suggesting that any combination of viruses had periods of co‐circulation and therefore the possibility of co‐infection.

### 3.2 Co‐Detection

The proportion of co‐detections for each virus are shown in Table 1, with co‐detection occurring overall in 4% of positive samples, ranging in the pre‐COVID‐19 era from 3% for Influenza virus to 39% for hBoV, with lowest co‐detection recorded for SARS‐CoV‐2 (0%). Co‐detections were much more common in children under 5 years, representing 9% of all infections, compared with 2% in older children and adults (Table S1). Interestingly, this differed in 2020/21, when there were no co‐detections in young children and co‐detections were more common in adults (data not shown). The number and proportion of co‐detections between pairs of viruses are shown by age in Figure 2B. Several viruses (ADV, hBoV and HCoV) had notably high proportions of co‐detection, particularly with RSV (especially among children aged under 6 months) and with HRV/ENV to a lesser extent. Co‐detections were much less common in age groups above 5 years old, with numbers increasing again above the age of 65. Regarding the 2020/21 season, the only virus to appear in co‐detection with SARS‐CoV‐2 was hBoV, as a single instance in the 65–74 age group.

**TABLE 1:** Detections and co‐detections by virus in hospitalised patients during study period.

Virus	Detections	Co‐detections	% co‐detections	95% confidence interval
Influenza A H1N1	1008	31	3%	(2%–4%)
Influenza A H3N2	2097	56	3%	(2%–3%)
Influenza A NT	22	0	0%	(0%–11%)
**Influenza A** ^a^	**3127**	**87**	**3%**	(2%–3%)
Influenza B Victoria	103	1	1%	(0%–4%)
Influenza B Yamagata	417	5	1%	(0%–3%)
Influenza B NT	45	7	16%	(7%–28%)
**Influenza B** ^a^	**565**	**13**	**2%**	(1%–4%)
Influenza NT	175	26	15%	(10%–21%)
**Influenza** ^a^	**3867**	**126**	**3%**	(3%–4%)
Adenovirus (ADV)	172	43	25%	(19%–32%)
Human bocavirus (hBoV)	215	84	39%	(33%–46%)
Non‐SARS human coronaviruses (HCoV)	869	167	19%	(17%–22%)
Human metapneumovirus (MPV)	574	37	6%	(5%–9%)
Human parainfluenzavirus (PIV)	176	11	6%	(3%–11%)
Rhino/enteroviruses (HRV/ENV)	2268	199	9%	(8%–10%)
Respiratory syncytial virus (RSV)	2608	258	10%	(9%–11%)
SARS‐CoV‐2	292	1	0%	(0%–2%)
**Total patients**	**10,570**	**455** ^b^	**4%**	(4%–5%)

*Note:* Confidence intervals were calculated according to the Jeffreys interval. Bold lines represent totals.

Abbreviation: NT = not typed.

^a^ Bold lines representing influenza totals were excluded from the final total.

^b^ Total patients with co‐detection is calculated as the sum of co‐detections and dividing by 2, such that each virus pair is not counted twice.

**FIGURE 3:**
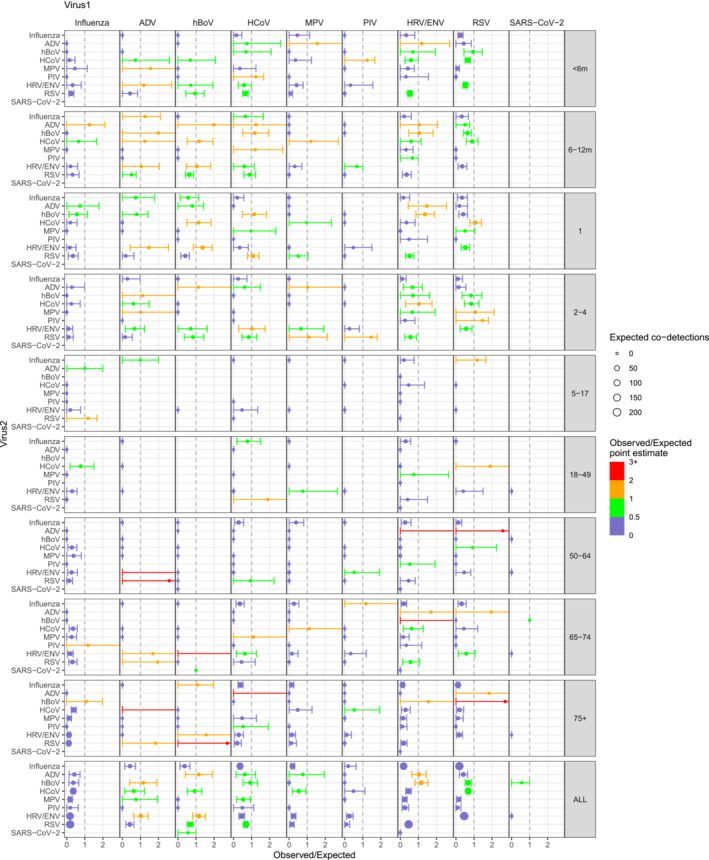
The point estimates and bootstrapped credible intervals of observed versus expected number of co‐detections for each pair of viruses by age group and all ages combined. The x‐axis represents the observed/expected ratio, with the point indicating the point estimate and the error bars the 2.5% and 97.5% percentile of the bootstrapped sample. The colour of the point and the error bar represent the observed expected ratio in the point estimate. The size of the point indicates the number of expected co‐detections, as a measure of the extent of co‐circulation. The y‐axis and the panel columns represent each pair of viruses, while each row of panels represents an age group.

Considering the weekly incidence of each virus in each age group, we calculated a crude measure for the expected number of co‐detections between each pair of viruses, accounting for individual viruses' circulation patterns. Examples of the epidemic curves over time for observed and expected co‐detections are shown for influenza and RSV (Figure S5), which show lower observed of co‐detection than expected and HCoV and RSV (Figure S5B) for which higher levels of co‐detection were observed. These values were summed over the whole time period to give total observed and total expected numbers of co‐detections (Table S2). From these, we then calculated the ratios of observed to expected co‐detections across the whole study period (Figure 3). The ratio was relatively low for influenza with all other viruses in any age group. There were comparatively more observed co‐detections in RSV, HRV/ENV and HCoV, particularly among children under 5 years. Looking at all age groups together, all combinations of HRV/ENV, ADV and hBoV tended to have high observed numbers of co‐detections compared to expected. Bootstrapping revealed that many of the very high point estimates of the observed/expected ratio, such as hBoV and HRV/ENV in the 65–74 age group, had very wide credibility intervals as they were based on small numbers of samples. The analysis excluding all seasons before 2014/15 led to similar results (Figure S6).

## 4 Discussion

We present here the analysis of an 11‐year active surveillance study of 9 respiratory viruses in a temperate region, targeting patients presenting with specific symptoms of acute respiratory illness and requiring hospitalisation. We describe independent seasonal patterns of hospitalisations linked to detections of single viruses and co‐detection of multiple viruses, highlighting the high rate of viral co‐detection in young children, and the lower than expected proportion of samples in which Influenza was co‐detected with other viruses.

Initially designed to track the Influenza season, the sampling period usually ran from November to March. Within this time window, we describe patterns of seasonality with high consistency for individual viruses across years but heterogeneous patterns between viruses. Influenza virus and RSV peaked in January–March and December–February, respectively. The Influenza A and B peaks in early January–February were consistent with the date of the peak estimated in Spain from WHO‐FluNet [4]. Based on a single year only, SARS‐CoV‐2 exhibited a January peak, which corresponds with the circulation of the alpha variant in the region at that time [35].

In 2017/18 and 2018/19, the data reported from the extended collection period suggests some substantial incidence outside of the November–March period for some viruses. First, we report an early start to HCoV detection, discordant with a previous suggestion that their circulation generally starts after December [1]. By opposition, the sharp earlier starts in September and long continuations for PIV and HRV/ENV, the year‐round incidence of ADV and hBoV, and the spring peak of MPV were consistent with previous studies from Spain [36] and a summary of temperate regions [1].

The appearance of co‐detections of a virus pair does not always correspond to periods of high co‐circulation with each virus separately. In order to systematically quantify this as coincidence or not, we compared the number of observed co‐detections to what would be expected under the assumption that viruses circulate independently. Our analysis suggests that, in hospitalised patients, some viruses, such as Influenza virus, and RSV in young children, appeared in co‐detection less often than would be expected given the level of co‐circulation, whereas some combinations such as HCoV and RSV appeared more than expected. Under the baseline hypotheses of independent circulation of the two viruses, higher levels of co‐detection than expected would be consistent with a hypothesis of synergistic interaction either in infection or hospitalisation, while lower levels are consistent with competition. We also note the strong age‐related patterns in these ratios, which could have different causes including age‐specific immune effects and sensitivities to viral interaction. In general, we report here a global number of observed co‐detections lower than expected, which is consistent with the majority of results in the literature suggesting predominantly competitive interactions between respiratory viruses [37].

Age had a substantial effect on rates of co‐detection, with many more co‐detections occurring in young children. This was also reflected in cohort studies from Normandy, France in 2019/20 [12] and India 2016–2018 [13]. Overall rates of co‐detection were comparable in the Indian study (3%) [13] which had a similar inclusion process to the current study own. The French study exhibited much higher co‐detection, with 14% of Influenza positive patients and 26% of RSV positive patients showing co‐detection [12], despite their including a much higher proportion of middle age groups, which exhibit relatively low co‐detection in our data. The differences between the present study and that by Petat et al. [12] could come from different enrolment strategies, as they were selected from primary care and based on clinical judgement of acute respiratory infection with recording of the range of symptoms [12] rather than a specified list of clinical criteria required before enrolment.

Time series of viral infections from Glasgow were analysed using statistical models and showed evidence for negative interaction of Rhinovirus with Influenza viruses A and B [14]. These results are consistent with our own, which found low co‐detection between HRV/ENV and combined Influenza. However, that study estimated positive interaction between Influenza virus and RSV, and between RSV and MPV [14], both contrasting with our own results. Another study of Influenza and RSV based only on a time series of tertiary hospitalisations in Vietnam used dynamic modelling to identify interactions, and again suggested either a positive or no interaction between these two viruses [15]. This origin of these differences from our own results is unclear and could come from differences in the manner of enrolment (the Glasgow study included primary and secondary hospitalisations in addition to tertiary), or continental differences (Vietnam experiences shallower seasonal spikes than Spain [38]). A negative effect on RSV infection of vaccination [39] or infection [40] with Influenza has been recorded in mouse models. However, among children infected with RSV, those where other viruses were co‐detected exhibited more severe infection [41]. Further analysis of our own data using dynamic modelling would reveal possible interactions with more nuance.

The results presented here should be interpreted in the light of the following limitations. First, the sampling period did not cover the whole year and was variable between seasons. Indeed, sampling was generally conducted during the months November–March, designated as the Influenza season, which means that infections occurring outside of this season were not observed, with particular implications for viruses whose high seasons continue outside the winter.

Second, the material used in the assays was changed from 2014/15 onwards, meaning that sensitivity increased across all viruses and for HRV/ENV and RSV particularly, with a corresponding increase in epidemic amplitude after this time. However, when the observed expected ratios were re‐analysed excluding the earlier seasons, we did not see a substantial change in results. In contrast to other similar cohort studies [12, 14], we used stringent clinical criteria for enrolment which were consistent over time, meaning our study populations were directly comparable between seasons.

Third, some viral species detected in the multiplex assay represent more than one circulating strain. The test intended for Rhinovirus cannot distinguish this from enteroviruses, while PIV subtypes 1–4, MPV types A and B, and different HCoVs are not differentiated. These different subtypes have distinct seasonality patterns in US studies, with HCoV 229E and HKU1 tending to peak in February, and NL63 and OC43 peaking in January [42] while in PIV, some subtypes are associated with winter and some with summer [43]. A study in Austria found that MPV incidence tends to be dominated by a particular subtype in each season, but that subtypes tend to exclude each other and then be replaced, with a shifting seasonality pattern [2]. Other viruses for which we did not test, such as paraechoviruses, as well as bacterial pathogens, may also be relevant in this population.

Fourth, the stringency of the inclusion criteria, which were intended to capture clear community‐acquired respiratory infections, may have missed relevant nosocomial circulation (as patients developing respiratory illness more than 7 days after hospital admission were excluded), may not be representative of mild disease, and may have missed those who were hospitalised for reasons of cardiovascular disease which had occurred as a direct consequence of acute viral respiratory infection [44].

Fifth, care should be taken in interpreting numbers of co‐detections as evidence for true positive or negative interactions. Lipsitch et al. [45] demonstrate that co‐detection studies are likely subject to selection bias, wherein the apparent effect of viral interaction in terms of infection can be confounded by an interaction with respect to the probability of showing symptoms and therefore being included in the sample. This is a valid point, and we emphasise that we cannot differentiate between an interaction in terms of the possibility of acquiring infection, and an interaction in terms of the possibility of seeking care. More critically, a modelling study by Domenech de Cellès et al. [46] dynamically modelled a two‐virus system and demonstrated that this type of analysis can be misleading in terms of the quantity of the interaction effect, or even the direction of the interaction effect if this interaction is not uniform throughout the virus infection and post‐infection period. Even in the absence of any interaction, there is the potential for co‐circulation of one virus to affect the incidence of another [47]. Furthermore, using co‐detection as a marker for co‐infection is a simplifying assumption since PCR positivity may persist long after symptoms and infectivity have lapsed [48]. Therefore, we provide the caveat that our results are interesting for comparison between age groups and pairs of viral species, but an identification of the true interactions between these pairs of viruses requires more explicit modelling of infection dynamics.

Finally, we highlight that the seasons since 2019/20 can be considered exceptional due to the COVID‐19 pandemic, which affected both the study sampling process, as well as the circulation of other pathogens, as a result of strong mitigation measures put in place (restriction in mobility, mask‐wearing, social distancing, hand washing prompts). However, as the seasonality and interaction of viruses with SARS‐CoV‐2 is relevant to this study, we have included these years regardless. Our results reflect these circumstances by the low incidence of other pathogens in 2020/21, as demonstrated in other studies reporting large reductions in circulation of non‐SARS‐CoV‐2 viruses [49]. We also report the exceptional absence of co‐detections in children in the 2020/21 season.

If the timing of the peak were representative of future seasonal SARS‐CoV‐2, in the absence of viral interference or non‐pharmaceutical interventions, COVID‐19 would peak simultaneously with Influenza virus and RSV, resulting in a ‘tripledemic’ with corresponding risk to health services and populations [50]. However, in the 2021/22 season in Spain, while there was overlap between these three viruses, the peaks occurred sequentially, with RSV peaking in early December, COVID‐19 in early January and Influenza virus in February [51], much later than any season since 2009 [52]. In 2022/23, the incidence of COVID‐19 across the EU was much lower than the previous years [53], while Influenza returned to pre‐pandemic levels [54], and RSV exhibited a strong early peak [55]. Further study is required to establish whether the disruption to normal seasonal patterns is a result of interaction with SARS‐CoV‐2. The introduction of both vaccination and/or passive immunisation against RSV from 2023 in Spain and elsewhere also has the potential to shift the viral peak [56].

The emergence of SARS‐CoV‐2 highlighted the increasing challenges to healthcare capacity and the need to better understand factors associated with seasonal demands. Detailed examination of the different viral causes of respiratory illness, their typical peaks, amplitudes and durations, as well identification of potential interactions, is key to quantifying and predicting the burden of respiratory virus‐associated hospitalisations.

## 5 Conclusions

Our analysis of large‐scale surveillance of patients hospitalised because of acute respiratory illness combined with multiplex assays over 2010–2021 reveal seasonal patterns in respiratory viruses. They also highlight extensive co‐detections of multiple viruses within the same patient, with strong variation across ages. Our results suggest differences between pairs of viruses in their tendency to co‐infect the same host. Further modelling work is required to ascertain these patterns and to assess whether they are representative only of hospitalised patients or more generally of outpatients or the community.

## Author Contributions

**George Shirreff:** formal analysis, methodology, visualization, writing – original draft. **Sandra S. Chaves:** data curation, funding acquisition, methodology. **Laurent Coudeville:** data curation, funding acquisition, methodology. **Beatriz Mengual‐Chuliá:** data curation, investigation. **Ainara Mira‐Iglesias:** data curation, investigation. **Joan Puig‐Barberà:** data curation, investigation. **Alejandro Orrico‐Sanchez:** data curation, investigation, project administration. **Javier Díez‐Domingo:** data curation, investigation. **Lulla Opatowski:** conceptualization, formal analysis, methodology, visualization. **F. Xavier Lopez‐Labrador:** conceptualization, data curation, investigation, methodology, writing – original draft.

## Conflicts of Interest

G.S. is funded by a Sanofi research grant through Institut Pasteur. S.S.C. and L.C. are employees of Sanofi and may hold shares in the company. J.D.D., A.M.I. and F.X.L.L. are employees at FISABIO foundation that have received funding from Sanofi and the Foundation for Influenza Epidemiology. A.M.I. has received fees for conferences/experts' meetings from Sanofi and for educational events from MSD. J.D.D. and his institution received grants from Sanofi and GSK related to RSV preventive strategies. J.D.D. acted as advisor for these immunisation strategies to Sanofi. L.O. received a research grant by Sanofi through Institut Pasteur. B.M.C. and J.P.B. declare no conflicts of interest.

### Peer Review

The peer review history for this article is available at https://www.webofscience.com/api/gateway/wos/peer‐review/10.1111/irv.70017.

## Supporting information


**Figure S1** Observed seasonal curves for each virus in each season, per capita in the total catchment population. The greyed out areas are those in which data were not collected, and the text underneath shows the dates of data collection in each season.
**Figure S2.** Mean incidence per week by virus category and age group. Each column is a different virus category and each row an age category.
**Figure S3.** Incidence for each type and subtype of Influenza viruses. The greyed‐out time points are those in which samples were not collected. The vertical dotted line indicates the beginning of each calendar year.
**Table S1.** Patients, positive and co‐detections by age group. The % co‐detections were calculated as a percentage of total positives, with confidence intervals calculated according to the Jeffreys interval.
**Table S2.** Numbers of observed vs expected co‐detections for each pair of viruses, in each age group. The calculated ratios are used to make the point estimates in Figure 3.
**Figure S4.** Estimated parameters in waveform analysis for each viral season, including ‘Peak week’: the timing of the peak in weeks relative to the final week in the calendar year; ‘Peak amplitude’: the maximum incidence per capita during the season; ‘Epidemic duration’: and the standard deviation of the peak width in number of weeks. The points represents the mean value of the posterior distribution, and the error bars the limits of the 95% credibility interval.
**Figure S5.** Timeline of observed vs expected numbers of co‐detections for (A) Influenza and RSV and (B) HCoV and RSV, aggregated by month. The red bars indicate the observed number of cases and the blue line the expected number assuming no interaction.
**Figure S6.** Following exclusions of data before the 2014/15 season, the point estimates and bootstrapped credible intervals of observed versus expected number of co‐detections for each pair of viruses by age group and all ages combined. The size of the point indicates the number of expected co‐detections, as a measure of the extent of co‐circulation. The colour indicates the ratio between observed and expected co‐detections in the point estimate, while the error bar indicates the 2.5% and 97.5% percentile of the boostrapped sample. Each row of panels represents an age group. ADV = adenovirus; hBoV = human bocavirus; HCoV = seasonal coronaviruses; MPV = human metapneumovirus; PIV = human parainfluenzavirus; HRV/ENV = rhino/enteroviruses; RSV = respiratory syncytial virus; SARS‐CoV‐2 = severe acute respiratory syndrome coronavirus 2.

## Data Availability

The anonymised, aggregated data that support the findings of this study are available on reasonable request from the corresponding author. The data are not publicly available due to privacy restrictions.

## Appendix A

### Valencia Hospital Surveillance Network for the Study of Influenza and Other Respiratory Viruses (VAHNSI)

Additional members of the VAHNSI group include Mario Carballido Fernández, Juan Mollar Maseres, Miguel Tortajada Girbés, Germán Schwarz Chávarri, Vicente Gil Guillén, Ramón Limón Ramírez, Empar Carbonell Franco, Angel Belenguer Varea, Concepción Carratalá Munuera and José Vicente Tuells Hernández.

**TABLE A1:** Clinical conditions used to identify admissions possibly associated with an influenza infection, reproduced from [22].

Patients ≥ 5 years of age	ICD 9 codes	ICD 10 codes
Acute respiratory infection	382·9; 460–466	J00–J06, J20–J22, H66·90
Acute myocardial infarction or acute coronary syndrome	410–411 and 413–414	I20–I25·9
Asthma	493–493·92	J45·2–J45·22, J45·9–J45·998, J44–J44·9
Heart failure	428–429·0	I50–I50·9; I51·4
Pneumonia and influenza	480–488	J09–J18
Chronic pulmonary obstructive disease	490, 491, 492, 496	J40–J44·9
Myalgia	729·1	M79·1
Metabolic failure (diabetic coma, renal dysfunction, acid–base disturbances, alterations to the water balance)	250·1– 250·3; 584–586; 276–277	E11·9, E10·9, E11·65, E10·65, E10·11, E11·01, E10·641, E11·641, E10·69, E11·00, E10·10, E11·69, N17·0, N17·1, N17·2, N17·8, N17·9, N18·1, N18·2, N18·3, N18·4, N18·5, N18·6M N18·9, N19, E87·0, E87·1, E87·2, E87·3, E87·4, E87·5, E87·6, E87·70, E87·71, E87·79, E86·0, E86·1
Altered consciousness, convulsions, febrile‐convulsions	780·01–780·02; 780·09; 780·31–780·32	R40·20, R40·4, R40·0, R40·1, R56·00, R56·01
Dyspnea/respiratory abnormality	786·0	R06·0, R06–R06·9
Respiratory abnormality	786·00	R06·9
Shortness of breath	786·05	R06·02
Respiratory abnormality NEC	786·09	R06·3, R06·00, R06·09, R06·83
Respiratory symptoms/chest symptoms	786·9	R06·89
Fever or fever unknown origin or non‐specified	780·6–780·60	R50, R50·9
Cough	786·2	R05
Sepsis, systemic inflammatory response syndrome	995·90–995·94	R65·10, R65·11, R65·20, A41·9

Patients 0–4 years of age	ICD 9 codes	ICD 10 codes
Acute upper or lower respiratory disease	382·9; 460 to 466	J00–J06, J20–J22
Dyspnoea, breathing anomaly, shortness of breath, tachypnoea	786·0; 786·00; 786·05–786·07; 786·09; 786·9	R06·0, R06, R06·9, R06·3, R06·00, R06·09, R06·83, R06·02, R06·82, R06·2, R06·89
Asthma	493–493·92	J45·2–J45·22, J45·9–J45·998, J44–J44·9
Pneumonia and influenza	480 to 488	J09–J18
Heart failure	428–429·0	I50–I50·9; I51·4
Myalgia	729·1	M79·1
Altered consciousness, convulsions, febrile convulsions	780·01–780·02; 780·09; 780·31–780·32	R40·20, R40·4, R40·0, R40·1, R56·00, R56·01
Fever or fever unknown origin or non‐specified	780·6–780·60	R50, R50·9
Cough	786·2	R05
Gastrointestinal manifestations	009·0; 009·3	A09·0; A09·9
Sepsis, systemic inflammatory response syndrome	995·90–995·94	R65·10, R65·11, R65·20, A41·9
