# Supplementary material for: Seasonality and Co‐Detection of Respiratory Viral Infections Among Hospitalised Patients Admitted With Acute Respiratory Illness—Valencia Region, Spain, 2010–2021
Source: Influenza Other Respir Viruses. 2024 Oct 22;18(10):e70017. doi: 10.1111/irv.70017 (PMC11496384; doi:10.1111/irv.70017)
Supplement: Supplementary file 1 — Figure S1 Observed seasonal curves for each virus in each season, per capita in the total catchment population. The greyed out areas are those in which data were not collected, and the text underneath shows the dates of data collection in each season. Figure S2. Mean incidence per week by virus category and age group. Each column is a different virus category and each row an age category. Figure S3. Incidence for each type and subtype of Influenza viruses. The greyed‐out time points are those in which samples were not collected. The vertical dotted line indicates the beginning of each calendar year. Table S1. Patients, positive and co‐detections by age group. The % co‐detections were calculated as a percentage of total positives, with confidence intervals calculated according to the Jeffreys interval. Table S2. Numbers of observed vs expected co‐detections for each pair of viruses, in each age group. The calculated ratios are used to make the point estimates in Figure 3. Figure S4. Estimated parameters in waveform analysis for each viral season, including ‘Peak week’: the timing of the peak in weeks relative to the final week in the calendar year; ‘Peak amplitude’: the maximum incidence per capita during the season; ‘Epidemic duration’: and the standard deviation of the peak width in number of weeks. The points represents the mean value of the posterior distribution, and the error bars the limits of the 95% credibility interval. Figure S5. Timeline of observed vs expected numbers of co‐detections for (A) Influenza and RSV and (B) HCoV and RSV, aggregated by month. The red bars indicate the observed number of cases and the blue line the expected number assuming no interaction. Figure S6. Following exclusions of data before the 2014/15 season, the point estimates and bootstrapped credible intervals of observed versus expected number of co‐detections for each pair of viruses by age group and all ages combined. The size of the point indicates the number of expected co‐d [file IRV-18-e70017-s001.pdf]

Supplementary Information

Additional data

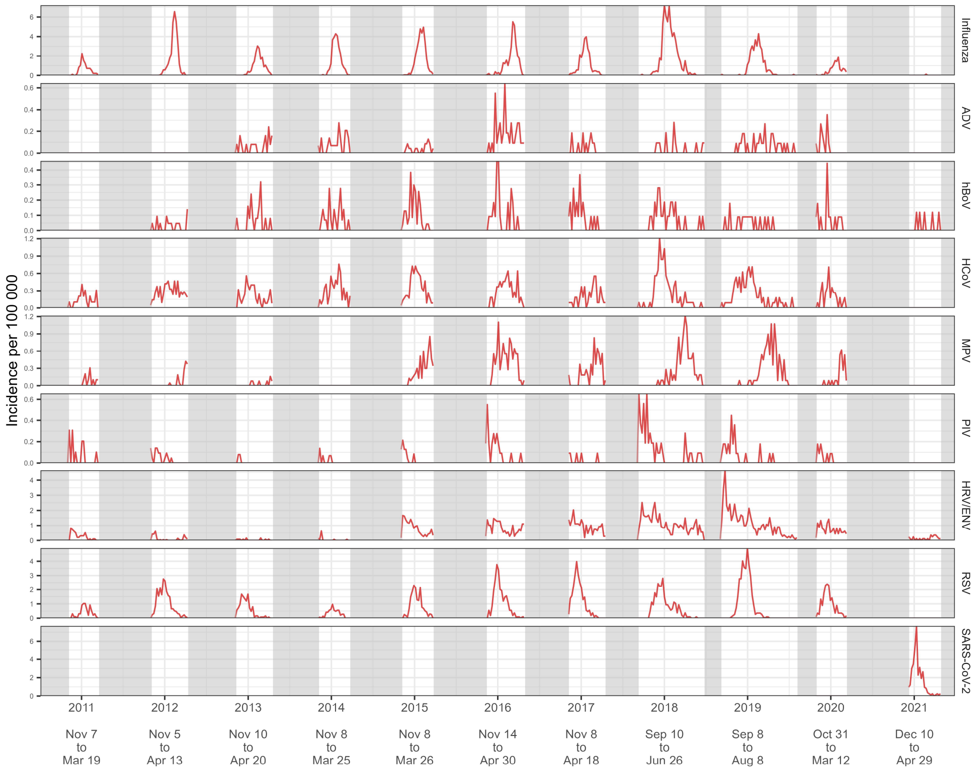

Supplementary Figure 1. Observed seasonal curves for each virus in each season, per capita in the total catchment population. The greyed out areas are those in which data were not collected, and the text underneath shows the dates of data collection in each season.

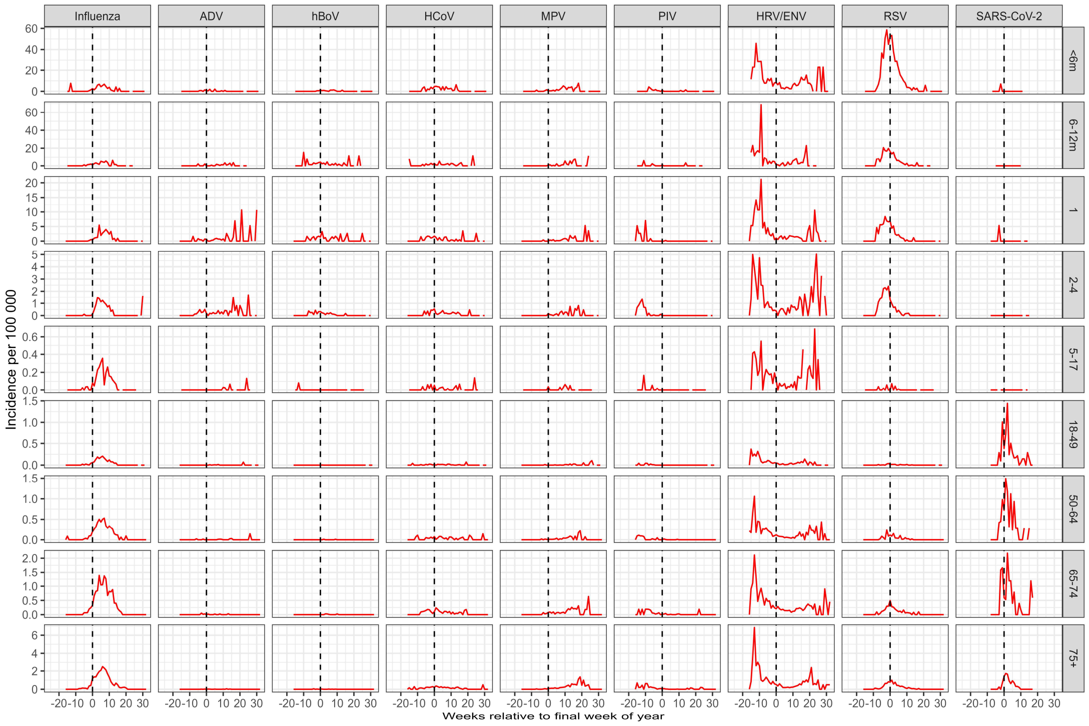

Supplementary Figure 2. Mean incidence per week by virus category and age group. Each column is a different virus category, and each row an age category.

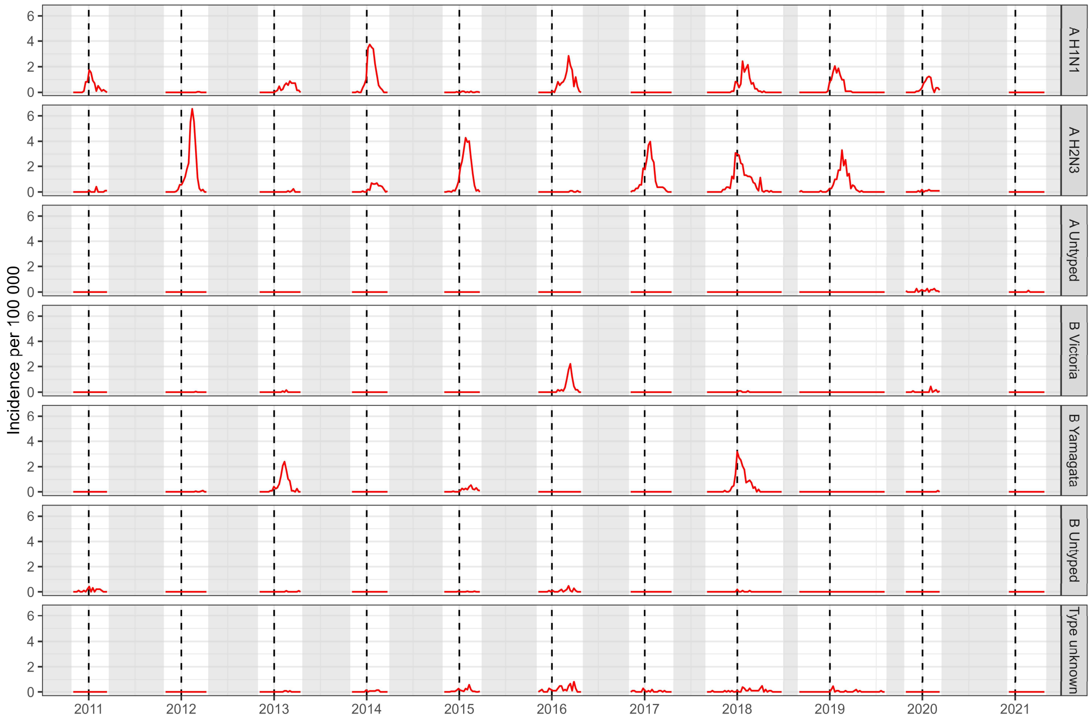

Supplementary Figure 3. Incidence for each type and subtype of Influenza viruses. The greyed-out time points are those in which samples were not collected. The vertical dotted line indicates the beginning of each calendar year.

Supplementary Table 1. Patients, positive and co-detections by age group. The % co-detections were calculated as a percentage of total positives, with confidence intervals calculated according to the Jeffreys interval.

Age group	Participants	Positives	Co-detections	% co-detections	95% confidence interval
<6m	2526	1589	132	8%	(7-10%)
6-12m	841	500	61	12%	(10-15%)
1	1040	590	69	12%	(9-14%)
2-4	1377	632	51	8%	(6-10%)
5-17	742	234	4	2%	(1-4%)
18-49	2254	679	12	2%	(1-3%)
50-64	3556	1097	20	2%	(1-3%)
65-74	4618	1432	38	3%	(2-4%)
75+	12030	3816	68	2%	(1-2%)
Age data missing	1	1	0	0%	(0-85%)
TOTAL	28985	10570	455	4%	(4-5%)

Supplementary Table 2. Numbers of observed vs expected co-detections for each pair of viruses, in each age group. The calculated ratios are used to make the point estimates in Figure 3.

Age group	Virus2	Virus1<br>Influenza	ADV	hBoV	HCoV	MPV	PIV	HRV / ENV	RSV	SARS-CoV-2
<6m	Influenza		0 / 1.1	0 / 0.9	1 / 6.3	2 / 4.4	0 / 0.4	3 / 9.3	10 /41.7	0 / 0
	ADV	0 / 1.1		0 / 0.2	1 / 1.4	1 / 0.6	0 / 0	3 / 2.5	4 / 9.3	0 / 0
	hBoV	0 / 0.9	0 / 0.2		1 / 1.5	0 / 0.5	0 / 0.1	1 / 1.4	8 / 8.4	0 / 0
	HCoV	1 / 6.3	1 / 1.4	1 / 1.5		1 / 2.8	1 / 0.8	7 /11.9	34 /51.5	0 / 0
	MPV	2 / 4.4	1 / 0.6	0 / 0.5	1 / 2.8		0 / 0.6	4 / 9.7	1 /15.2	0 / 0
	PIV	0 / 0.4	0 / 0	0 / 0.1	1 / 0.8	0 / 0.6		1 / 3.2	0 / 4.9	0 / 0
	HRV / ENV	3 / 9.3	3 / 2.5	1 / 1.4	7 /11.9	4 / 9.7	1 / 3.2		52 /98.3	0 / 0
	RSV	10 /41.7	4 / 9.3	8 / 8.4	34 /51.5	1 /15.2	0 / 4.9	52 / 98.3		0 / 0
	SARS-CoV-2	0 / 0	0 / 0	0 / 0	0 / 0	0 / 0	0 / 0	0 / 0	0 / 0	
6-12m	Influenza		2 / 1.6	0 / 2.4	2 / 3	0 / 1.1	0 / 0	1 / 4.7	3 / 9.3	0 / 0
	ADV	2 / 1.6		3 / 1.5	1 / 0.8	0 / 0.2	0 / 0.4	2 / 1.9	2 / 3.9	0 / 0
	hBoV	0 / 2.4	3 / 1.5		5 / 4.3	0 / 1.3	0 / 0.6	7 / 6.7	14 /22	0 / 0
	HCoV	2 / 3	1 / 0.8	5 / 4.3		1 / 0.8	0 / 0	3 / 5	13 /14.6	0 / 0
	MPV	0 / 1.1	0 / 0.2	0 / 1.3	1 / 0.8		0 / 0	1 / 3.1	0 / 2.4	0 / 0
	PIV	0 / 0	0 / 0.4	0 / 0.6	0 / 0	0 / 0		1 / 1.5	0 / 1.3	0 / 0
	HRV / ENV	1 / 4.7	2 / 1.9	7 / 6.7	3 / 5	1 / 3.1	1 / 1.5		6 /17.3	0 / 0
	RSV	3 / 9.3	2 / 3.9	14 /22	13 /14.6	0 / 2.4	0 / 1.3	6 /17.3		0 / 0
	SARS-CoV-2	0 / 0	0 / 0	0 / 0	0 / 0	0 / 0	0 / 0	0 / 0	0 / 0	
1	Influenza		2 / 2.7	4 / 7.1	1 / 4.9	0 / 2.6	0 / 0	1 / 5.8	4 /11.9	0 / 0
	ADV	2 / 2.7		2 / 2.5	0 / 1.7	0 / 0.1	0 / 0	4 / 2.7	1 / 5.3	0 / 0
	hBoV	4 / 7.1	2 / 2.5		7 / 6.1	0 / 1.3	0 / 0.2	14 /10.3	6 /14.9	0 / 0
	HCoV	1 / 4.9	0 / 1.7	7 / 6.1		1 / 1	0 / 0.2	2 / 5.8	15 /14	0 / 0
	MPV	0 / 2.6	0 / 0.1	0 / 1.3	1 / 1		0 / 0	0 / 3.2	1 / 2	0 / 0
	PIV	0 / 0	0 / 0	0 / 0.2	0 / 0.2	0 / 0		1 / 2.1	0 / 0.8	0 / 0
	HRV / ENV	1 / 5.8	4 / 2.7	14 /10.3	2 / 5.8	0 / 3.2	1 / 2.1		13 /24.9	0 / 0
	RSV	4 /11.9	1 / 5.3	6 /14.9	15 /14	1 / 2	0 / 0.8	13 /24.9		0 / 0
	SARS-CoV-2	0 / 0	0 / 0	0 / 0	0 / 0	0 / 0	0 / 0	0 / 0	0 / 0	
2-4	Influenza		1 / 3.5	0 / 3	1 / 3.9	0 / 2.6	0 / 0	1 / 9.4	1 / 9.3	0 / 0
	ADV	1 / 3.5		1 / 0.9	2 / 3.2	1 / 1	0 / 0.2	4 / 6	1 / 6.6	0 / 0
	hBoV	0 / 3	1 / 0.9		0 / 1.5	0 / 1.1	0 / 0	3 / 4.3	7 / 8.4	0 / 0
	HCoV	1 / 3.9	2 / 3.2	0 / 1.5		0 / 1.3	0 / 0.1	5 / 4.9	9 /10.7	0 / 0
	MPV	0 / 2.6	1 / 1	0 / 1.1	0 / 1.3		0 / 0	2 / 3	2 / 1.8	0 / 0
	PIV	0 / 0	0 / 0.2	0 / 0	0 / 0.1	0 / 0		1 / 3.9	1 / 0.7	0 / 0
	HRV / ENV	1 / 9.4	4 / 6	3 / 4.3	5 / 4.9	2 / 3	1 / 3.9		10 /17.8	0 / 0
	RSV	1 / 9.3	1 / 6.6	7 / 8.4	9 /10.7	2 / 1.8	1 / 0.7	10 /17.8		0 / 0
	SARS-CoV-2	0 / 0	0 / 0	0 / 0	0 / 0	0 / 0	0 / 0	0 / 0	0 / 0	
5-17	Influenza		1 / 1	0 / 0	0 / 0.9	0 / 0.7	0 / 0.2	1 / 5.1	1 / 0.8	0 / 0
	ADV	1 / 1		0 / 0	0 / 0	0 / 0	0 / 0	0 / 0	0 / 0	0 / 0
	hBoV	0 / 0	0 / 0		0 / 0	0 / 0	0 / 0	0 / 0.5	0 / 0	0 / 0
	HCoV	0 / 0.9	0 / 0	0 / 0		0 / 0	0 / 0.1	1 / 2.2	0 / 0.3	0 / 0
	MPV	0 / 0.7	0 / 0	0 / 0	0 / 0		0 / 0	0 / 0.4	0 / 0	0 / 0
	PIV	0 / 0.2	0 / 0	0 / 0	0 / 0.1	0 / 0		0 / 1	0 / 0	0 / 0
	HRV / ENV	1 / 5.1	0 / 0	0 / 0.5	1 / 2.2	0 / 0.4	0 / 1		0 / 0.7	0 / 0
	RSV	1 / 0.8	0 / 0	0 / 0	0 / 0.3	0 / 0	0 / 0	0 / 0.7		0 / 0
	SARS-CoV-2	0 / 0	0 / 0	0 / 0	0 / 0	0 / 0	0 / 0	0 / 0	0 / 0	
18-49	Influenza		0 / 1.3	0 / 0	4 / 5.2	0 / 1.1	0 / 0	3 /10.2	0 / 3.9	0 / 0
	ADV	0 / 1.3		0 / 0	0 / 0.3	0 / 0	0 / 0	0 / 0.1	0 / 0	0 / 0
	hBoV	0 / 0	0 / 0		0 / 0	0 / 0	0 / 0	1 / 0.2	0 / 0	0 / 0
	HCoV	4 / 5.2	0 / 0.3	0 / 0		0 / 0.3	0 / 0	0 / 3.2	2 / 1.1	0 / 0
	MPV	0 / 1.1	0 / 0	0 / 0	0 / 0.3		0 / 0	1 / 1.3	0 / 0	0 / 0
	PIV	0 / 0	0 / 0	0 / 0	0 / 0	0 / 0		0 / 0.9	0 / 0	0 / 0
	HRV / ENV	3 /10.2	0 / 0.1	1 / 0.2	0 / 3.2	1 / 1.3	0 / 0.9		1 / 2.5	0 / 1.2
	RSV	0 / 3.9	0 / 0	0 / 0	2 / 1.1	0 / 0	0 / 0	1 / 2.5		0 / 0
	SARS-CoV-2	0 / 0	0 / 0	0 / 0	0 / 0	0 / 0	0 / 0	0 / 1.2	0 / 0	
50-64	Influenza		0 / 1.1	0 / 0.3	4 /14.3	3 / 7.9	0 / 0.9	4 / 15	2 /14.7	0 / 0
	ADV	0 / 1.1		0 / 0	0 / 0.1	0 / 0.1	0 / 0	1 / 0.3	1 / 0.4	0 / 0
	hBoV	0 / 0.3	0 / 0		0 / 0.1	0 / 0	0 / 0	0 / 0	0 / 0	0 / 0.8
	HCoV	4 /14.3	0 / 0.1	0 / 0.1		0 / 0.7	0 / 0.2	0 / 3.4	2 / 2.1	0 / 0
	MPV	3 / 7.9	0 / 0.1	0 / 0	0 / 0.7		0 / 0	0 / 3.8	0 / 0.9	0 / 0
	PIV	0 / 0.9	0 / 0	0 / 0	0 / 0.2	0 / 0		1 / 1.9	0 / 0.4	0 / 0
	HRV / ENV	4 /15	1 / 0.3	0 / 0	0 / 3.4	0 / 3.8	1 / 1.9		3 / 6.6	0 / 1.6
	RSV	2 /14.7	1 / 0.4	0 / 0	2 / 2.1	0 / 0.9	0 / 0.4	3 / 6.6		0 / 0
	SARS-CoV-2	0 / 0	0 / 0	0 / 0.8	0 / 0	0 / 0	0 / 0	0 / 1.6	0 / 0	
65-74	Influenza		0 / 1	0 / 0.9	7 /20.2	3 /11.2	1 / 0.8	5 /26.3	7 /22.3	0 / 0
	ADV	0 / 1		0 / 0	0 / 0.1	0 / 0.2	0 / 0	1 / 0.6	1 / 0.5	0 / 0
	hBoV	0 / 0.9	0 / 0		0 / 0.1	0 / 0	0 / 0	1 / 0.1	0 / 0	1 / 1
	HCoV	7 /20.2	0 / 0.1	0 / 0.1		2 / 1.8	0 / 0.8	4 / 6.4	2 / 4.5	0 / 0
	MPV	3 /11.2	0 / 0.2	0 / 0	2 / 1.8		0 / 0.3	1 / 6.5	0 / 2.4	0 / 0
	PIV	1 / 0.8	0 / 0	0 / 0	0 / 0.8	0 / 0.3		1 / 3.1	0 / 0.9	0 / 0
	HRV / ENV	5 /26.3	1 / 0.6	1 / 0.1	4 / 6.4	1 / 6.5	1 / 3.1		5 / 8.8	0 / 0.4
	RSV	7 /22.3	1 / 0.5	0 / 0	2 / 4.5	0 / 2.4	0 / 0.9	5 / 8.8		0 / 0
	SARS-CoV-2	0 / 0	0 / 0	1 / 1	0 / 0	0 / 0	0 / 0	0 / 0.4	0 / 0	
75+	Influenza		0 / 1	2 / 1.8	20 /52.5	4 /32.4	0 / 2.9	8 /64	9 /80.6	0 / 0
	ADV	0 / 1		0 / 0	1 / 0.3	0 / 0.3	0 / 0	0 / 0.5	1 / 0.5	0 / 0
	hBoV	2 / 1.8	0 / 0		0 / 0.4	0 / 0.1	0 / 0.1	1 / 0.6	2 / 0.7	0 / 0
	HCoV	20 /52.5	1 / 0.3	0 / 0.4		3 / 6.3	1 / 1.9	5 /17.4	4 /19.9	0 / 0
	MPV	4 /32.4	0 / 0.3	0 / 0.1	3 / 6.3		0 / 1.1	3 /20	1 / 8.4	0 / 0
	PIV	0 / 2.9	0 / 0	0 / 0.1	1 / 1.9	0 / 1.1		1 / 9.6	0 / 3.1	0 / 0
	HRV / ENV	8 /64	0 / 0.5	1 / 0.6	5 /17.4	3 /20	1 / 9.6		6 /32.9	0 / 0.1
	RSV	9 /80.6	1 / 0.5	2 / 0.7	4 /19.9	1 / 8.4	0 / 3.1	6 /32.9		0 / 0
	SARS-CoV-2	0 / 0	0 / 0	0 / 0	0 / 0	0 / 0	0 / 0	0 / 0.1	0 / 0	
ALL	Influenza		6 /14.3	6 /16.5	40 /111.2	12 /63.9	1 / 5.2	27 /149.9	37 /194.5	0 / 0
	ADV	6 /14.3		6 / 5.2	5 / 7.9	2 / 2.7	0 / 0.7	15 /14.7	11 /26.6	0 / 0
	hBoV	6 /16.5	6 / 5.2		13 /14	0 / 4.4	0 / 1	28 /24.1	37 /54.4	1 / 1.8
	HCoV	40 /111.2	5 / 7.9	13 /14		8 /15.1	2 / 4.1	27 /60.3	81 /118.6	0 / 0
	MPV	12 /63.9	2 / 2.7	0 / 4.4	8 /15.1		0 / 2.1	12 /51.1	5 /33.2	0 / 0
	PIV	1 / 5.2	0 / 0.7	0 / 1	2 / 4.1	0 / 2.1		7 /27.1	1 /12	0 / 0
	HRV / ENV	27 /149.9	15 /14.7	28 /24.1	27 /60.3	12 /51.1	7 /27.1		96 /209.8	0 / 3.4
	RSV	37 /194.5	11 /26.6	37 /54.4	81 /118.6	5 /33.2	1 /12	96 /209.8		0 / 0
	SARS-CoV-2	0 / 0	0 / 0	1 / 1.8	0 / 0	0 / 0	0 / 0	0 / 3.4	0 / 0	

Waveform analysis: methods and results

The number of observed incident cases, in all age groups and including co-detections of virus *v* in week *t* is represented as $Inc_{v,t}$. To analyse the seasonal patterns for each virus season, the waveform incidence ($WInc_{v,t}$) was modelled after [33] using a waveform model with a unimodal bell curve function of the form:

$$WInc_{v,t} = N_s \frac{A_{vs}}{\cosh^2 \frac{t - \varphi_{vs}}{\sigma_{vs}}}$$

where *cosh* is the hyperbolic cosine function and N_s is the size of the catchment population in that season. The timing and shape of each viral seasonal epidemic was defined by its peak week (φ_{vs}), its peak amplitude (A_{vs}), and the epidemic duration (σ_{vs}), which were estimated using a Bayesian approach calculating the Poisson likelihood of the number of $Inc_{v,t}$ given $WInc_{v,t}$. Statistical inference was conducted using *modMCMC* from the *FME* package in *R* [34,61].

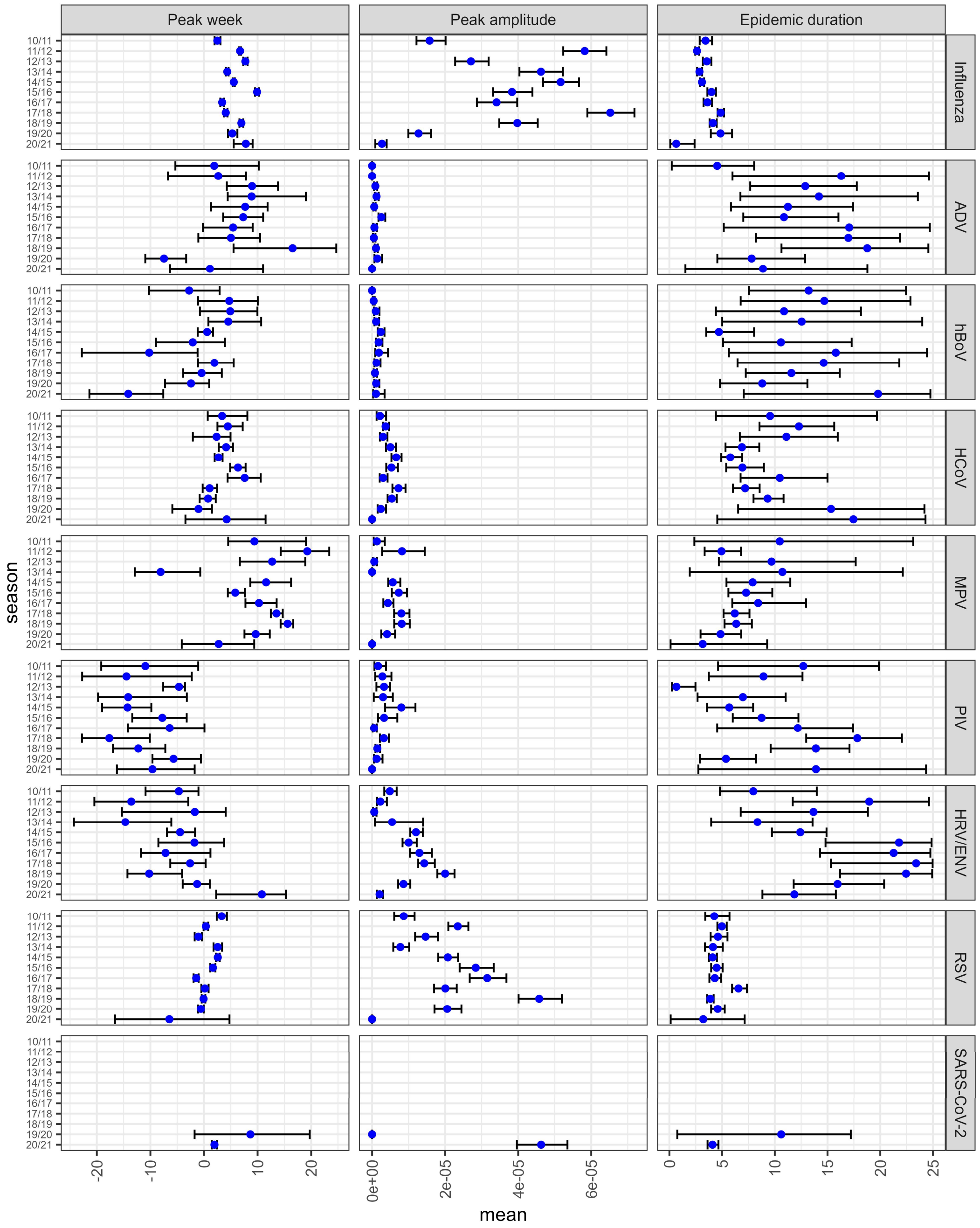

Supplementary Figure 4. Estimated parameters in waveform analysis for each viral season, including "Peak week": the timing of the peak in weeks relative to the final week in the calendar year; "Peak amplitude": the maximum incidence per capita during the season; "Epidemic duration": and the standard deviation of the peak width in number of weeks. The points represents the mean value of the posterior distribution, and the error bars the limits of the 95% credibility interval.

Example epidemic curves of observed vs expected co-detections

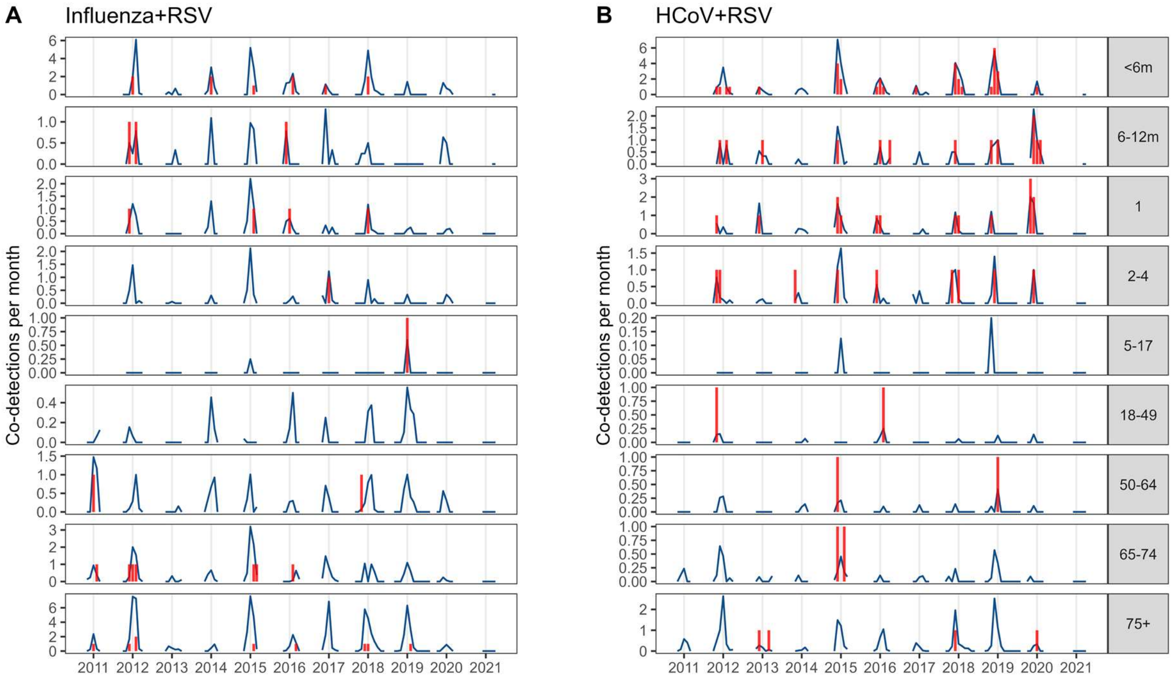

Supplementary Figure 5. Timeline of observed vs expected numbers of co-detections for A) Influenza and RSV and B) HCoV and RSV, aggregated by month. The red bars indicate the observed number of cases and the blue line the expected number assuming no interaction.

Observed expected ratios in data since 2014/15

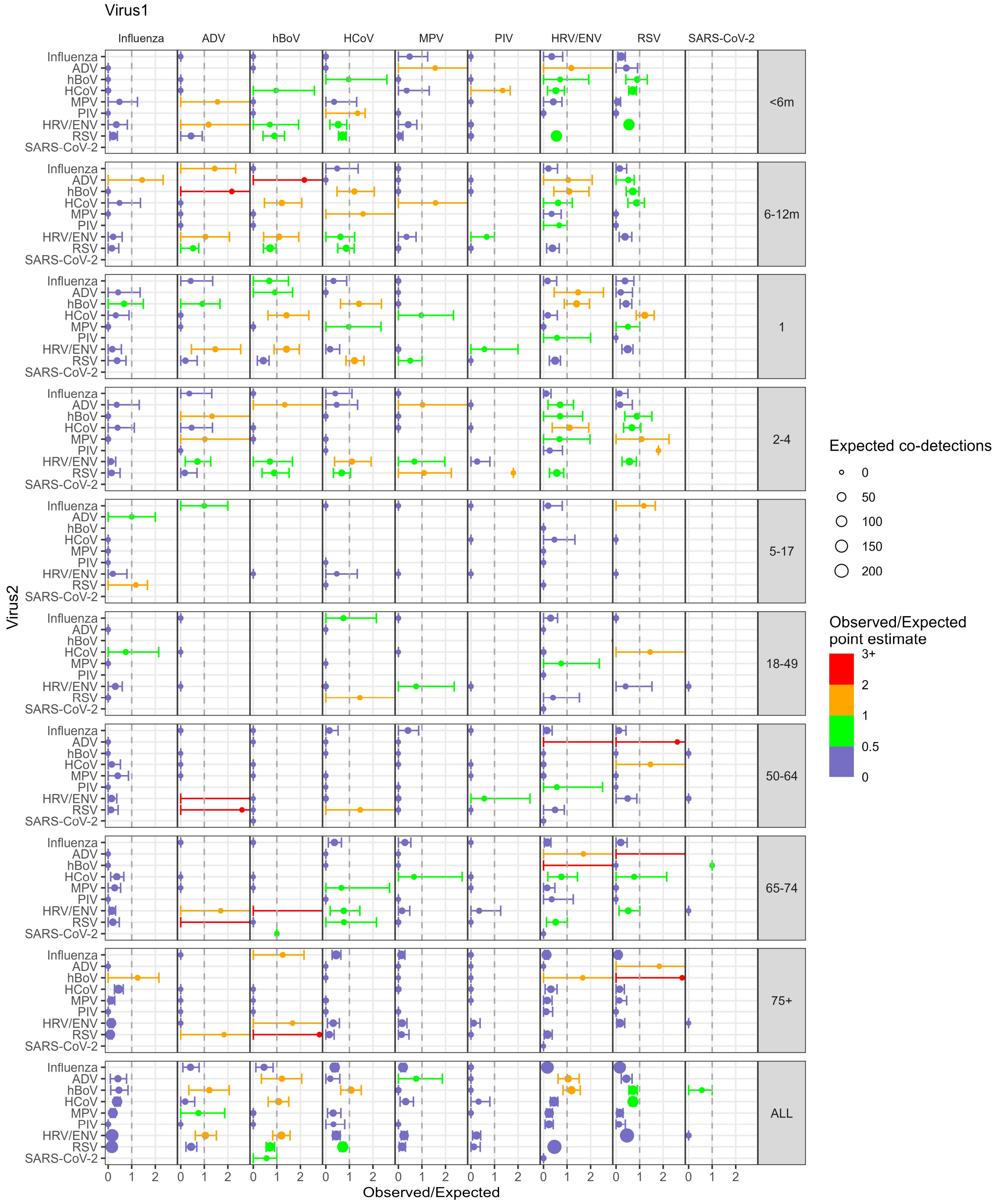

Supplementary Figure 6. Following exclusions of data before the 2014/15 season, the point estimates and bootstrapped credible intervals of observed vs expected number of co-detections for each pair of viruses by age group and all ages combined. The size of the point indicates the number of expected co-detections, as a measure of the extent of co-circulation. The colour indicates the ratio between observed and expected co-detections in the point estimate, while the error bar indicates the 2.5% and 97.5% percentile of the boostrapped sample. Each row of panels represents an age group. ADV = Adenovirus; hBoV = Human bocavirus; HCoV=Seasonal coronaviruses; MPV=Human metapneumovirus; PIV=Human parainfluenzavirus; HRV/ENV=Rhino/enteroviruses; RSV=Respiratory Syncytial Virus; SARS-CoV-2=Severe Acute Respiratory Syndrome Coronavirus 2.
